# Regulation of Glycogen Synthase Kinase 3β Functions by Modification of the Small Ubiquitin-Like Modifier

**DOI:** 10.2174/1874091X00802010067

**Published:** 2008-04-13

**Authors:** Eun Jeoung Lee, Sung Hee Hyun, Jaesun Chun, Sung Hwa Shin, Kwang Hum Yeon, Min Kyoung Kwak, Tae Yoon Park, Sang Sun Kang

**Affiliations:** 1 School of Science Education, Chungbuk National University, Gaeshin-dong, Heungdok-gu, Cheongju, Chungbuk, 361-763, Republic of Korea.; 2 Department of Pre-medicine, Eulji University School of Medicine, Daejeon 301-832, Republic of Korea; 3 Department of Biology Education, Korea National University of Education, Chongwon, Chungbuk 363-791, Republic of Korea; 4 Graduate School of Education, Yonsei University, 134 Shinchon-dong, Seodaemun-gu, Seoul 120-749, Republic of Ko-rea

**Keywords:** GSK 3β, SUMOylation, cell apoptosis, protein stability, subcellular localization, kinase activation

## Abstract

Modification of the Small Ubiquitin-like Modifier (SUMO) (SUMOylation) appears to regulate diverse cellular processes, including nuclear transport, signal transduction, apoptosis, autophagy, cell cycle control, ubiquitin-dependent degradation and gene transcription. Glycogen synthase kinase 3β (GSK 3β) is a serine/threonine kinase that is thought to contribute to a variety of biological events, including embryonic development, metabolism, tumorigenesis, and cell death. GSK 3β is a constitutively active kinase that regulates many intracellular signaling pathways by phosphorylating substrates such as β-catenin. We noticed that the putative SUMOylation sites are localized on K^292 ^residueof ^291^FKFPQ^295^ in GSK 3β based on analysis of the SUMOylation consensus sequence. In this report, we showed that the SUMOylation of GSK 3β occurs on its K^292^ residue, and this modification promotes its nuclear localization in COS-1. Additionally, our data showed that the GSK 3β SUMO mutant (K292R) decreased its kinase activity and protein stability, affecting cell death. Therefore, our observations at first time suggested that SUMOylation on the K^292^ residue of GSK 3β might be a GSK 3β regulation mechanism for its kinase activation, subcellular localization, protein stability, and cell apoptosis.

## INTRODUCTION

SUMO (Small Ubiquitin-like Modifier) modification (SUMOylation) of proteins, especially of transcriptional regulators and nuclear pore proteins has been described [1]. SUMO is a new covalent modification leading to attachment of SUMO to specific lysine residues of target proteins [2, 3]. SUMO represents a class of ubiquitin-like proteins conjugated, like ubiquitin, by a set of enzymes to cellular proteins [5]. However, SUMOylation does not promote protein degradation and distinct enzymes are involved in SUMOylation. For SUMOylation of substrate proteins, SAE1/SAE2 heterodimer acts as an E1 enzyme in mammals (Aos1/Uba2 in yeast), and Ubc9 acts as an E2 SUMO-conjugating enzyme [6]. The mammalian PIAS (protein inhibitor of activated STAT) family, Ran Bp (Ran binding protein) 2 and the polycomb PC2 repressor, have recently been shown to function as E3-type SUMO ligases [7-10]. The analysis of many SUMOylation substrates indicates that it occurs at a particular sequence, thus the specificity of SUMO conjugation might be conferred by recognition of this sequence by the thioester-linked Ubc9–SUMO conjugate [11-13]. Lysine residues targeted for SUMOylation are often found within specific sequences, such as its consensus sequence [5, 14].

Glycogen synthase kinase 3β (GSK 3β) is a constitutively active serine/threonine kinase that regulates many intracellular signaling pathways by phosphorylating substrates such as β-catenin [15, 16]. The phosphorylation of β-catenin by GSK 3β is facilitated by the scaffold protein axin and is inhibited either by GSK 3-binding protein (GBP), also known as Frat (Frequently rearranged in advanced T-cell lymphomas), or Dishevelled. The phosphorylation of β-catenin by GSK 3β, resulting in its ubiquitin-mediated proteolysis, is the axis of the canonical Wnt signaling pathway. Thus, GSK 3β is thought to contribute to a variety of biological events, such as embryonic development, metabolism, tumorigenesis, and cell death [17]. The function of GSK 3β is also regulated through phosphorylation by other protein kinases including Akt, which is a serine/threonine kinase that is activated by phosphatidylinositol 3-kinase (PI3K) signaling and phosphorylates GSK 3β on S^9^, thereby inactivating it [18, 19].

During our investigation of the Akt-GSK 3β signal transduction pathway, we noticed the putative SUMOylation site (K^292 ^in ^291^FKFPQ^295^) in GSK 3β using consensus motif computer analysis (http://www.abgent.com/doc/sumoplot). This finding led us to evaluate whether GSK 3β is SUMOylated or not. And then, we observed the SUMOylation of GSK 3β with *in vitro *or *in vivo* SUMOylation assay. Additionally, we identified that the SUMOylation site of GSK 3β is the K^292^ residue using site directed mutagenesis analysis. We also characterized the biological significances of GSK 3β SUMOylation using a GSK 3β kinase assay, confocal microscopy and FACS analysis. Therefore, in this article, our data suggest that SUMOylation in GSK 3β is one of the regulation mechanisms for kinase activity, protein stability, and nuclear localization, as well as affecting cell apoptosis. Even though it is unclear how SUMOylation of GSK 3β occurs in the cell, we suggest here that SUMOylation on the K^292^ residue of GSK 3β seems to be a new mechanism for its functional regulation.

## MATERIALS AND METHODS

### Cell Culture

COS-1 was purchased from ATCC (Manassas, VA, USA). Media and supplements were obtained from GIBCO (Grandisland, NY, USA). The cell line was maintained in Dulbecco’s Modified Essential Medium (DMEM) containing 10% heat inactivated (for 30 min at 56℃) fetal bovine serum (FBS), 100 U potassium penicillin/ml, 100 μg streptomycin/ml, 2 mM glutamine and 20 mM sodium bicarbonate. The cells were incubated at 5% CO_2_, 95 % humidity and 37℃ and growth medium changed every 3 days. SUMO fusion protein was obtained from Calbiochem (Grandisland, NY). Wild type human GSK 3β was purchased in Ha- or GST-tagged mammalian expression vector (GeneCopoeia Co. CA, USA).

### Antibodies

Monoclonal antibody against the Ha epitope or GST was purchased from Santa Cruz Biotech. Inc. (Santa Cruz, CA, USA). Antibodies against GSK 3β or human Tau specific antibody were purchased from Santa Cruz Biotech. Inc. (Santa Cruz, CA, USA). actin antibody was purchased from Cell Signaling Technology, Inc. (Cell Siganling Co. MA, USA). Antibodies against Tau 422 Ser phosphor was purchased from Calbiochem. (La Jolla, CA, Germany). Antibodies against SUMO-1 was purchased from ABGENT ( San Diego, CA, USA).

### Site-Directed Mutagenesis of GSK 3β

To generate GSK 3β, K292R, and K340R (UP; 5’-aac tac aca gaa ttt aGG ttc cct caa att aag gca-3’, Down; 5’-aat ttg agg gaa CCt aaa ttc tgt gta gtt tgg gtt-3) and (UP; 5’- cgg gac cca aat gtc aGG cta cca aat ggg cga gac-3’, Down 5’- ccc att tgg tag CCt gac att tgg gtc ccg taa ttc-3) from GSK 3β were used [20] with a “Chameleon” double-stranded site-directed mutagenesis kit (Stratagene, CA, USA), according to the manufacturer’s instructions. Every mutation was confirmed by DNA sequencing.

### GSK 3β Expression Vector Transfection and Purification

For mammalian expression, Ha-GSK 3β wt or GSK 3β SUMO mutant construct were transfected into COS-1 cells using the lipofectin transfection method (Gibco-BRL Co). Transfected cells (2x10^7^) were lysed in RIPA lysis buffer. Anti-Ha monoclonal antibody was incubated with 1000 μl of pre-cleaned cell lysate and precipitated with protein A agarose beads. The beads were then washed three times with excess cell lysis buffer and the final pellet used for the immuno assay to detect SUMOyaltion. Western blots were performed with anti-SUMO-1 antibody todetect the presence of SUMO [3, 21]. To detect the phosphorylation of GSK 3β T^216^ residue, an anti-216 Tyr phospho Ab (La Jolla, CA, Germany) was used.

### Double Immunofluorescence Microscopy

COS-1 cells were plated at a low confluence (~30%) on two-well Lab-Tek Permanox slides (NalgeneNunc International, Naperville, IL) and transiently transfectedwith Ha –GSK 3β wt or Ha-GSK 3β SUMO mutant (K292R) plasmid usingthe lipofectamine procedure.Cells were starved for 36 h and subsequently treated with10% calf serum for 15 h. At no time did cell confluency exceed 60%. Cells were fixed, permeabilized, and processed for indirectdouble immunofluorescence microscopy as described previously, withminor modifications. Cells were blocked innormal goat and diluted (1:30) in PBS for 15 min, then incubatedwith affinity-purified, anti- antibodies at 1:150 dilutionin combination with a 1:1000 dilution of murine SUMO-1 monoclonalantibodies (ABGENT, San Diego, CA, USA), anti- GSK 3β S^9^ residue, an anti-9 Ser phospho Ab (purchased from Cell Siganling Co. MA, USA), or Ha monoclonal antibodies (Santa Cruz, CA, USA) at room temperature for 1-2 h on a rocking platform. Washed slides were then incubated for 1 h atroom temperature with 1:150 dilutions of both anti-rabbit fluoresceinisothiocyanate-conjugated secondary antibody (Molecular ProbesInc. OR, USA) and Texas red-conjugated goat anti-rabbit secondaryantibody (Molecular Probes Inc. CA, USA). Slides were washed and thenmounted with Vectashield mounting medium (Vector LaboratoriesInc. CA, USA) and examined using Leica TCS SPⅡ AOBS in The Core Facility of Chungbuk National University [20].

### Expression and Purification of Recombinant Proteins

GST tagged GSK 3β wt was purchased from GeneCopia TM and its SUMO mutant (K292R) was cloned with the same primer set used for generation of mammalian SUMO mutants. GST tagged protein was expressed in *Escherichia coli* BL21 and purified with GST-agarose beads according to the manufacturer’s instruction (Amersham Biosciences Co). Purified proteins were used for the SUMOylation assay substrate protein.

### In vitro SUMO-1 Conjugation Assay

SUMO-1 conjugation assay was performed *in vitro* using the SUMO assay kit purchased from Corgen Inc (Taipei, Taiwan), according to the manufacturer’s recommended protocol. One microgram of purified GST-tagged GSK 3β mutants were mixed with 250 ng of Ubc9, 125 ng of Aos1/Uba2, with or without 2 µg of SUMO-1, then incubated for 2 hr at 30°C in the presence of 50 mM Tris [pH 7.5], 5 mM MgCl2, with or without (for the negative control) 2 mM ATP in a 20 µl volume. Reactions were stopped with SDS-PAGE sample buffer and SUMO conjugates separated by SDS-PAGE and analyzed by western blotting using the mouse monoclonal antibody against SUMO-1 to detect GSK 3β [3]. The relative optical density (OD) was measured by image analysis of the dried SDS-PAGE gel using the Fuji Image Quant software (Fujifilm, Tokyo, Japan), according to the manufacturer’s instructions.

### GSK 3β Kinase Assay

Human Tau protein was purchased from Biomol (CA, USA). GSK 3β kinase assays were performed for 30 minutes at 30 °C in a 25-µl reaction volume containing [20 mM HEPES, pH 7.2, 10 mM MgCl_2_, 10 mM MnCl_2_, 1 mM dithiothreitol, 0.2 mM EGTA,and 1 μg phosphatidylserine], and 1µg human Tau protein as a GSK 3β substrate protein. The phosphorylated Tau protein was detected using a Western blot with Tau S^422^ phosphor specific antibody, purchased from Calbiochem (La Jolla, CA, Germany).

### Protein Stability Experiments

COS-1 cells (2.5 × 10^5^ cells per well) in 10cm plates were transfected with 1.0 μg of expression vector with Ha-GSK 3β wt or SUMO mutant plasmid. The medium was replaced with medium containing 200 μg/ml cycloheximide 36 h after transfection (0-h time point). Cell lysates were harvested at 0, 8, 16, and 24 h then analyzed by immunoprecipitation and Western blotting using anti-Ha antibodies, and assayed in five time repeats. The relative optical density (OD) was measured by image analysis of the dried SDS-PAGE gel with the Fuji Image Quant software (Fujifilm, Tokyo, Japan), according to the manufacturer’s instructions.

### FACS Analysis

Ha-GSK 3β (wt), Ha-GSK 3β SUMO mutant (K292R), or pcDNA vector was transfected and the rate of apoptosis measured by Annexin V-PE apoptosis detection kit I (BD Biosciences, CA, USA), according to the manufacturer’s instructions. Transfected Cells were washed twice in cold PBS and then resuspended in Binding buffer (0.01 M Hepes/NaOH (pH 7.4) 0.14 M Nacl, 2.5 mM CaCl_2_). 1X10^5 ^cells in 100 μl were transferred to 5ml culture tube and adde 5 μl of Annexin V-PE and 5 μl of 7-Amino-actinomycin D. The cells were vortexed gently and incubated for 15 min at 25 °C in the dark. 400 μl of binding buffer was added to each tube. Within 1 hr, FACS was performed on a Coulter Epics Elite equipped with a gated amplifier and upgraded with enhanced system performance in The Core Facility of Chungbuk National University [20].

## RESULTS

### SUMOylation of GSK 3β *In Vitro* and *In Vivo*

Using computer analysis of the SUMOylation consensus sequence from GSK 3β, two sites were found in the C-terminal domain (K^292 ^in ^291^FKFPQ^295^), which is near by the Axin and FRAT binding domains (262-299aa), as shown in Fig. (**1A**) [22, 23]. Therefore, we predicted that GSK 3β is one of the SUMO modified (SUMOylation) proteins [3, 13]. To test our prediction, we constructed GSK 3β SUMO mutant (K292R) which is indicated in Fig. (**1A**) below, by site directed mutagenesis. Both the GSK 3β wild type (wt) and SUMO mutant (K292R) was inserted into Ha tagged expression vector (for eukaryotic cell) or GST fusion expression vector (for prokaryotic cell).

**Fig. (1). GSK 3β functional domain and SUMOylation:**
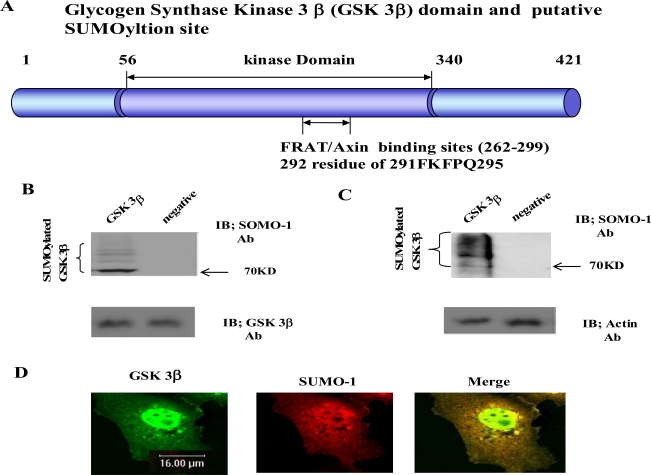
The Glycogen synthase kinase 3β (GSK 3β) functional domains (its protein kinase and FRAT/Axin binding domain) and the putative SU-MOylation site (K^292^ in ^291^FKFPQ^295^) is indicated **(A)**. GSK 3β SUMO mutant (K292R) was constructed by site directed mutagenesis. GSK 3β SUMO mutant (K292R) was inserted into GST fusion (for bacteria) and Ha fusion (for cell line) expression vectors. **(B)** GSK 3β wild type (wt) protein that was purified from E. coli was incubated with a SUMOylation assay kit (See Material and method). For the negative control, the same assay conditions were used without ATP (right lane). A western blot of the same sample was performed with GSK 3β monoclonal antibody to monitor the protein amount in the experiment (at bottom). SUMOylated GSK 3β, as several high molecular weight protein bands, was indicated. **(C)** A western bolt of the immunopurified GSK 3β from COS-1 was performed using the SUMO-1 specific antibody. SUMOylation of GSK 3β was detected as high molecular weight protein bands, as indicated (left lane). For the negative control, an unrelated mouse antibody was used (right lane). To monitor the total protein amount to be used in the cell lysates, the western blot was per-formed with actin monoclonal antibody (bottom). **(D)** Confocal microscopic analysis of endogenous GSK 3β wt (green color) and SUMO-1 (red color). GSK 3β was detected in both the cytoplasm and nuclear region. The SUMO-1 modification proteins were mainly detected in the nuclear region (yellow color). All the figures in this article represent results from three experiments repeated independently.

Initially we performed the *in vitro* SUMOylation assay using GST-GSK 3β fusion protein purified from *E. coli* to determine whether SUMOylation of GSK 3β occurred, as described in the Materials and Methods section [21], using a SUMO assay for GSK 3β wt without ATP as a negative control (Fig. **1B**, right lane). The western blot of each sample was performed using GSK 3β antibody to monitor the protein amount in the experiment (at bottom). As shown in Fig. (**1B**), SUMOylated GSK 3β was detected, including several high molecular weight protein bands (left lane), suggesting that GSK 3β is one of SUMOylation proteins *in vitro*.

To confirm GSK 3β SUMOylation in the cell, we performed a western bolt of the immunopurified GSK 3β from COS-1 cells with SUMO-1 specific antibody, as described in the Materials and Methods section [21]. As shown in Fig. (**1C**), SUMOylation of GSK 3β was detected as high molecular weight protein bands (left lane), similar to the results in Fig. (**1B**). An unrelated mouse antibody was used as a negative control (right lane). To monitor the total protein amount to be used in the cell lysates, western blot was performed with actin monoclonal antibody (Fig. **1C**, bottom). Therefore, the results depicted in Fig. (**1C**) also suggest that GSK 3β was SUMOylated in COS-1 cells, which is consistent with the results in Fig. (**1B**).

Confocal microscopy observation of COS-1 cells with GSK 3β or SUMO_1 specific antibody was used to observe whether endogenous GSK 3β undergoes SUMOylation in the cell. As shown in Fig. (**1D**), GSK 3β, which was detected in both the cytoplasm and the nucleus, was merged with SUMO-1 in the nucleus. These results suggest that endogenous GSK 3β in COS-1 was modified by SUMO-1, which is consistent with Fig. (**1B** and **C**). Interestingly, SUMOylation of GSK 3β seems to be required for its nuclear localization (Fig. **1D**). Therefore, overall, these results (Fig. **1A**, **B**, **C**, and **D**) suggest that GSK 3β is one of the SUMOylation proteins.

### SUMOylation Sites in GSK 3β

As shown in Fig. (**1A**), we predicted that K^292 ^in (^291^FKFPQ^295^) of GSK 3β was the putative SUMOylation sites based on consensus sequence analysis. To test our prediction, we performed the SUMOylation assay *in vitro* with GSK 3β SUMO mutant (K292R). We used GST fusion GSK 3β (wt) or GSK 3β SUMO mutant, as the substrate protein. Western blotting of the same sample was performed with GST monoclonal antibody to monitor the amount of protein in the experiment (at bottom). We also performed the SUMOylation assay using GST fusion, GSK 3β K292R mutant protein, and did not observe the SUMOylation of each mutant protein (data not shown). As shown in Fig. (**2A**), the SUMOylation of GSK 3β SUMO mutant was not detected, whereas that of GST fusion GSK 3β (wt) was observed. Therefore, our data suggested that K^292 ^in (^291^FKFPQ^295^) of GSK 3β is the putative SUMOylation sites.

**Fig. (2). SUMOylation Site in GSK 3β:**
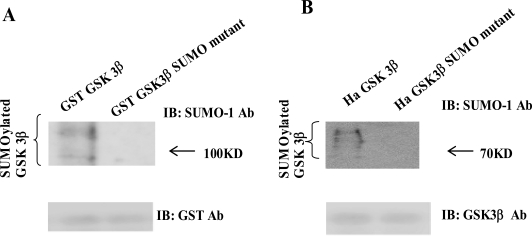
**(A)** The purified GST-GSK 3β wt or GST-GSK 3β SUMO mutant (K292R) fusion protein was used as the substrate protein in the SUMOy-lation assay as described in the Materials and Methods section. The SUMOylation of GSK 3β wt was detected as a high molecular weight protein band (left lane), whereas its SUMO mutant was totally inhibited, as shown (right lane). **(B)** Ha –GSK 3β wt or Ha –GSK 3β SUMO mutant was transfected to COS-1 cells and immunoprecipitated with Ha mouse monoclonal antibody. The immunoprecipitants were sub-jected to the western bolt with SUMO-1, as described in the Materials and Methods section. The SUMOylation of GSK 3β wt was indicated as several high molecular weight protein bands (left lane), whereas its SUMO mutant was totally inhibited (right lane). To monitor the GSK 3β protein expression, the immunoprecipitants were subjected to the western bolt with GSK 3β polyclonal antibody (bottom).

To confirm our observation further, we performed a western bolt using the immunopurified Ha-GSK 3β wt or Ha -GSK 3β SUMO mutant (K292R) from COS-1 cells (Fig. **2B**), using the SUMO-1 specific antibody. To monitor the total protein amount to be used in the cell lysates, western blot was performed using GSK 3β polyclonal antibody (bottom) (Fig. **2B**). As shown in Fig. (**2B**), SUMOylation of GSK 3β wt was detected as high molecular weight protein bands (right lane), whereas GSK 3β SUMO mutant (K292R) was not. Thus, as indicated in Fig. (**2A** and **B**), these data suggest that SUMOylation of GSK 3β occurs on K^292^ residuein ^291^FKFPQ^295^.

### Confocal Microscopic Analysis with GSK 3β SUMO Mutant

**Fig. (3). Confocal microscopic analysis of GSK 3β wt or its SUMO mutant:**
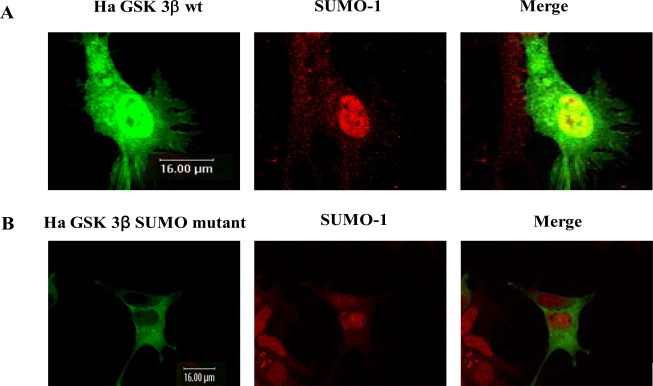
Confocal microscopic analysis of transfected Ha –GSK 3β wt **(A)**, Ha –GSK 3β SUMO mutant (K292R) **(B)** was performed to determine whether it merged with SUMO-1 (red color). All Ha –GSK 3β constructs were shown as green color. The transfected Ha -GSK 3β wt (de-tected in both the cytoplasm and the nucleus) merged (yellow) with SUMO-1 in the nucleus **(A)**. The transfected Ha –GSK 3β SUMO mutant was detected in the cytoplasm, but not in the nucleus **(B)**. The SUMO-1 modification proteins were mainly detected in the nuclear region (**B** middle lane). GSK 3β SUMO mutant in which the SUMOylation site was eliminated was not merged with SUMO-1 in the nucleus (**B** right lane).

Next, we indented to determine the biological significance of SUMOylation on K^292^ of GSK 3βBecause the confocal microscopy results in Fig. (**1D**) suggested that SUMOylation of GSK 3β occur in the nuclear region, we first determined whether SUMOylation of GSK 3β affects its subcellular localization. We performed the confocal microscopic analysis with Ha -GSK 3β wt or the SUMO mutant construct, as described in Fig. (**1A**). The transfected Ha –GSK 3β wt or Ha –GSK 3β SUMO mutant (K292R) was detected as green and the SUMO-1 position was detected as red using fluorescence microscopy (Fig. **3**). Similar to the results shown in Fig. (**1D**), SUMO-1 signals were mainly detected in the nucleus (Fig. **3A** middle lane). Ha -GSK 3β wt was observed in both the cytoplasm and nucleus, similar to the results shown in Fig. (**1D**). However, the merged between GSK 3β with SUMO-1 (yellow color) was detected in the nucleus and not the cytoplasm (Fig. **3A** right lane), suggesting that SUMOylation of GSK 3β is related to its nuclear subcellular localization. This result also supports the idea that GSK 3β is one of the SUMOylation proteins, consistent with the results presented in Fig. (**1D**).

Surprisingly, Ha –GSK 3β SUMO mutant was mainly detected in the cytoplasm, not the nucleus, and was not merged (yellow color) with SUMO-1 in the nucleus (Fig. **3B** right lane). Therefore, these results suggested that SUMOylation of GSK 3β is required for its nuclear localization, consistent with Fig. (**1D** and **3A**). Further, consistent with the results shown in Fig. (**2**), this result (Fig. **3B**) again confirmed that K^292^ of GSK 3β is SUMOylation sites by the confocal microscopic analysis.

### SUMOylation of GSK 3β is Required for its Kinase Activation

**Fig. (4). Kinase activity of GSK 3β wt or its SUMO mutant:**
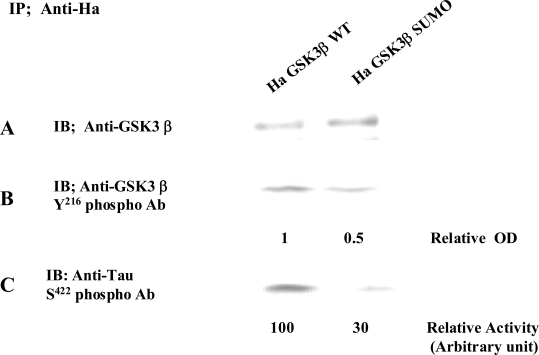
The immunopurified Ha -GSK 3β wt or its SUMO mutant (K292R) protein with Ha Ab from COS-1 was immunoblotted with GSK 3β **(A)** or Anti- GSK 3β Tyr 216 phospho Ab polyclonal antibody **(B)**. The relative optical density (OD), as determined by image analysis with the Fuji Image Quant software, is indicated below. The GSK 3β kinase activity was measured using human Tau protein as a substrate **(C)**. S422 residue phosphorylation of human Tau protein was detected with its specific antibody. The relative GSK 3β activity by image analysis with the Fuji Image Quant software is indicated below. Results shown are one of five repeated experiments

To further define the biological significance of GSK 3β SUMOylation, we compared the kinase activity of GSK 3β with that of the GSK 3β SUMO mutant. Each Ha –GSK 3β wt or its SUMO mutant (K292R) expression vector was transfected into COS-1 cells, and immunoprecipitated with Ha monoclonal Ab. To monitor the expression of Ha -GSK 3β wt or GSK 3β SUMO mutants, western blot was performed with an anti-GSK 3β antibody (Fig. **4A**). Because it has been reported that 216 tyrosine residue phosphorylation of GSK 3β is required for its activation [17, 24], we monitored it with its 216 tyrosine phosphorylation specific antibody (Fig. **4B**). As shown in Fig. (**4B**), we observed that 216 tyrosine phosphorylation of the Ha -GSK 3β SUMO mutant was reduced compared to that of Ha –GSK 3β wt (Fig. **3B**), even though the expressed protein amount was almost same (Fig. **4A**). Next, we measured the kinase activity with its substrate protein Tau, because Ser 422 of human Tau is phosphorylated by GSK 3β [25, 26]. As shown in Fig. (**4C**), we observed that the kinase activity of the GSK 3β SUMO mutant was reduced to half that of the GSK 3β wt, consistent with its 216 tyrosine phosphorylation result (Fig. **3B**). Taken together, our data suggest that SUMOylation of GSK 3β is also required for not only its 216 tyrosine phosphorylation, but also its kinase activity. To eliminate cell line specificity, we performed the experiment using NIH 3T3 cells and obtained the same results (data not shown).

### The Effect ofGSK 3β SUMOylation onProtein Stability

**Fig. (5). Protein stability of GSK 3β wt and its SUMO mutant:**
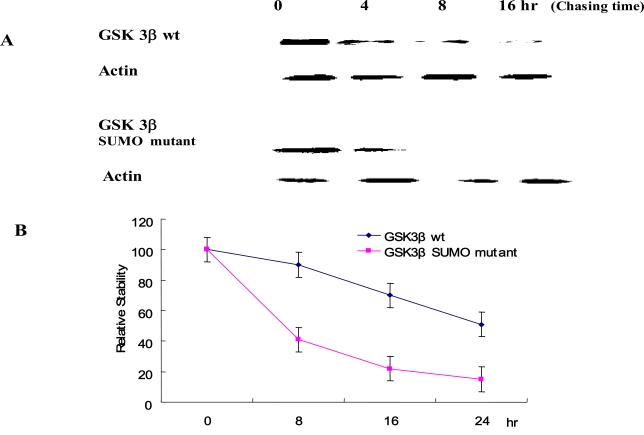
Ha –GSK 3β or GSK 3β SUMO mutant (K292R) was transfected into COS-1 cells and the cells treated with cyclohexamide. The GSK 3β proteins were chased for the indicated time periods. Ha–GSK 3β proteins were immunoprecipitated with a polyclonal anti- Ha antibody and subjected to SDS-PAGE followed by western blotting with a monoclonal GSK 3β antibody **(A)**. To monitor the protein amount, an equal amount of cell lysate was subjected to western blotting with an actin antibody. Results shown are one of five repeated experiments. Quantifi-cation of the pulse-chase experiment is shown in **(B)** by image analysis with the Fuji Image Quant software.

To evaluate the effect of SUMOylation on GSK 3β protein stability, we performed the pulse-chase experiments as described in the Materials and Methods section. Each Ha -GSK 3β (wt or SUMO mutant) expression vector was transfected into COS-1 cells and immunoprecipitated with Ha monoclonal Ab following cyclohexamide treatment (Fig. **5A**). GSK 3β proteins were chased for the indicated time periods (0, 8, 16, 24 hr), and then immunoprecipitated with a polyclonal anti- Ha antibody and subjected to SDS-PAGE followed by western blot with GSK 3β antibody. To control the protein amount, we monitored the actin in each sample by western blotting (Fig. **5A**). The quantification of the pulse-chase experiment, as determined by image analysis of the dried SDS-PAGE gel using the Fuji Image Quant software, is shown in Fig. (**5B**). As shown in Fig. (**5**), the protein stability of the GSK 3β wt was twice that of the GSK 3β SUMO mutant, suggesting that SUMOylation on the K^292^ of GSK 3β seems necessary for protein stability.

### The Effect of GSK 3β SUMOylation onCell Viability

We measured cell viability using FACS analysis to determine whether SUMOylation on K^292^ of GSK 3β influenced cell viability. As shown in Table **1**, the FACS results indicate that the GSK 3β SUMO mutant (K292R) increased the cell survival rate significantly compared to the Ha -GSK 3β wt or pcDNA vector alone. Thus, GSK 3β SUMO mutant was only less effective on apoptosis than the Ha -GSK 3β wt or pcDNA vector alone (Table **1**).

In summary, our results indicate that SUMOylation on K^292^ of GSK 3β regulates protein stability, kinase activity, nuclear localization, and cell apoptosis. Therefore, our observations suggest that SUMOylation is a GSK 3β functional modification for its regulation.

## DISCUSSION

Because GSK 3β is a multi-functional protein kinase that may regulate biological functions, such as embryonic development, metabolism, tumorigenesis, and cell death, by regulation of many intracellular signaling pathways through phosphorylation substrates, such as β-catenin, the characterization of GSK 3β modification is essential to understand its function and regulation [15-17].

To understand the modification of GSK 3β, we first tested whether GSK 3β is a SUMOylated protein or not. After computer analysis of the SUMOylation motif indicated that GSK 3β seems to be SUMOyalted, we confirmed that GSK 3β is one of the SUMOylated proteins by conducting a SUMOylation assay, and through confocal microscopy analysis (Fig. **1D** and **3**). After confirming that GSK 3β is a SUMOylated protein, we intended to know which lysine residues of GSK 3β are SUMOylated. To do so, we performed site directed mutagenesis using computer SUMOyaltion consensus motif analysis (Fig. **1A**) and found that SUMOylation occurs on the K^292^ in ^291^FKFPQ^295 ^of GSK 3β (Fig. **2** and **3**). Next, to identify the biological significance of GSK 3β SUMOylation, we compared its protein kinase activity, subcellular localization, and stability with those of the GSK 3β SUMO mutant and found that SUMOylation of GSK 3β affected its biological roles, including protein kinase activity, subcellular localization, stability, and cell viability. (Fig. **3-5**, Table **1**). Therefore, although the molecular mechanism underlying GSK 3β SUMOylation and its regulation remain unknown, our data suggest that SUMOylation occurs on GSK 3β protein as a novel posttranslational step for GSK 3β functional regulation. Further studies are essential to elucidate the molecular mechanism involved in GSK 3β mediated signal transductions through SUMOylation. In particular, it will be important to determine exactly when and where SUMOylation of GSK 3β occurs, as well as how it is regulated, because GSK 3β is involved in a variety of human diseases, including cancer and Alzheimer’s [22, 23, 27-29].

It has been reported that SUMO-1 (but not SUMO-2, 3 and 4) monomerically conjugates the ε amino group of the K residue in SUMO-1 acceptor consensus sequences [1, 2, 12, 30]. As shown in Fig. (**1B** and **C**), however, the SUMOylation of GSK 3β wt was detected as several high molecular weight protein bands (~200 kD), which indicates that multiple SUMOylation on several other K residues of GSK 3β occurred. Even though SUMO forms a homo or hetero dimmer in the cell, SUMOyaltion by other SUMO isotypes (SUMO-2, 3 and 4) may be excluded in this experiment, because SUMO-1 monoclonal antibody was used. We noticed several K residues in GSK 3β where multiple SUMOylation was possible on GSK 3β by SUMO-1 [31]. However, it is presently unclear why SUMOylation of GSK 3β occurs as high molecular weight protein bands *in vivo and in vitro* in SUMOylation assays conducted using SUMO-1 specific antibody.

Our identification of and K^292^ residue of GSK 3β as targets for the majority of SUMOylation will help determine whether it plays a role in the normal β catenin degradation assembly. Phosphorylation of β-catenin by GSK 3β has been shown to be facilitated by the scaffold protein axin and is inhibited either by GSK3-binding protein (GBP), also known as Frat (Frequently rearranged in advanced T-cell lymphomas), or by Dishevelled [22, 23, 27-29]. Thus, basing on our results, which indicate that the GSK 3β SUMO mutant promotes cell survival (Table **1**), it seems that the GSK 3β SUMO mutant facilitates its dissociation with axin but facilitates association with GBP/Frat or Dishevelled (thus staying in the cytoplasm), resulting in inhibition of phosphorylation of β-catenin and its ubiquitin-mediated proteolysis. SUMOylation on the K^292^ residue of GSK 3β seems to regulate not only protein interaction with Frat/Axin, but also its activation. SUMOylation on the K^292^ residue of GSK 3β also seems to be one of the cell death signals (Table **1**), resulting from the increase of its protein stability and nuclear localization (Fig. **1D**, Fig. **3**). Therefore, we speculated that because GSK 3β is one of the major effectors of Wnt signaling [15, 16, 17], SUMOylation on the K^292^ residue of GSK 3β affected cell survival (Table **1**). Even though it is unknown how SUMOylation of GSK 3β regulates phosphorylation of its substrates, such as β-catenin, we are now evaluating the effect of GSK 3β SUMOylation on phosphorylation of β-catenin, its stability and Wnt signaling.

It is presently unknown how GSK 3β is transported in the nuclear region, where SUMOylation occurs. Since GSK 3β is known as a cytoplasmic and nuclear protein [32, 33], GSK 3β seems to have its own nuclear localization sequence (NLS) or nuclear export sequence (NES). We did not notice any NLS or NES consensus sequence homology around the SUMOylation site, K^292^ [34-36]. However, we can not rule out the possibility that our GSK 3β SUMO mutant (K292R) influences its own NLS or NES function directly or indirectly, because of the diversity of the NLS or NES consensus sequence [34, 35].

Even though it has been reported that accumulation of GSK 3β in the nucleus is related to the cell cycle, we do not presently know whether our results regarding GSK 3β nuclear localization and its SUMOylation is related to cell cycle regulation [32, 33]. It has been demonstrated that Akt, which is activated by phosphatidylinositol 3-kinase (PI3K) signaling, phosphorylates GSK 3β on S^9^, thereby inactivating it [18, 19]. Our preliminary data suggested that phosphorylation of GSK 3β on S^9^ seems to be unrelated to SUMOylation (data not shown), however it is still unknown what types of cell signals stimulate SUMOylation of GSK 3β on K^292^. Even though we performed the experiment using NIH 3T3 cells and observed the same results as described above, due to the possibility of cell line specificity, we can not say for certain that SUMOylation of GSK 3β on K^292^ regulates its kinase activity, subcellular localization, protein stability, and apoptosis in normal cells. Further, we can not rule out the possibility that the mutation on K^292^ of GSK 3β affect directely on its protein kinase activity, regardless of its SUMOylation.

E1 enzyme, E2 SUMO-conjugating enzyme, and E3 SUMO ligases involved in GSK 3β SUMOylation still remain to be characterized and there mechanism identified. Therefore, more studies will be required to elucidate the physiological significance of SUMOylation on K^292^ residue of GSK 3β. Because lysine serves as the attachment site for several modifications, including ubiquitination, acetylating, and methylamine [37], it seems important that multiple SUMOylation on other lysine residues of GSK 3β, which is trigged by SUMOylation on the K^292 ^residue, also plays a role by antagonizing other post-translational modifications. Because the half-life of the GSK 3β SUMO mutant was reduced compared to that of the GSK 3β wt (Fig. **5**), we speculated that the GSK 3β SUMO mutant is more ubiqutinized than that of the GSK 3β wt. However, the relationship between SUMOyaltion and ubiquitinization (or other modification) of GSK 3β also remains to be characterized.

Currently, four SUMO subtypes (SUMO-1, 2, 3 and 4) have been identified [5, 14, 38]. The SUMOylation consensus sequences for each subtype are known to be the same as SUMO-1 (Φ**K**xE/D, where Ф represents L, I, V or F and x is any amino acid) [11-13]. However, the K^292^ residuein ^291^FKFPQ^295^of GSK 3β does not belong to the exact SUMOylation consensus sequences (Ф**K**xE/D, where Ф represents L, I, V or F and x is any amino acid). We do not presently know how great an effect the difference between the GSK 3β amino acid sequence and the consensus sequences has on SUMOylation. Therefore, the SUMO subtypes that modify GSK 3β should be characterized, and the SUMOylation subtype that controls each specific GSK 3β function should be determined.

In conclusion, point mutagenesis analysis suggested that SUMOylation of GSK 3β occurs on its K^292^ residue, which is overlapped with the Frat or Axin binding domain by SUMO-1 (Fig. **1A**), and SUMOylation of GSK 3β promotesits nuclear localization and protein stability, resulting in the stimulation of cell apoptosis. Overall, our results suggest that SUMOylation on the K^292^ residue of GSK 3β may be a new GSK 3β regulation mechanism for kinase activity, subcellular localization, protein stability, and apoptosis. Our findings may also provide a new intervention clue to cure the many human diseases caused by the abnormal regulation of GSK 3β mediated signal transductions, such as cancers, diabetes, and Alzheimer’s disease.

## Figures and Tables

**Table 1:** Cell viability of GSK 3β wt and its SUMO mutant.

**GSK 3β construct **	**Rate of apoptosis (%) Be FACS**
Ha-GSK3 β (wt)	25 +/- 3
Ha-GSK3 β (SUMO mutant)	5 +/- 2
pcDNA (vector only)	10 +/- 2

Ha -GSK 3β (wt) or (SUMO mutant, K292R) or pcDNA vector was transfected and the rate of apoptosis measured by FACS. Ha -GSK 3β SUMO mutant (K292R), which was dominantly localized in the cytoplasm, promoted cell survival (but not cell apotosis) when compared to GSK 3 β wt constructs. Results shown are the average of five repeated experiments. For details, see the Materials and Methods section.
